# The Sunday Fund's Success

**Published:** 1920-08-07

**Authors:** 


### The Hospital

The Workers' Newspaper of Administrative Medicine and Institutional Life, Administration, National Insurance and Health.

No. 1783, Vol. LXVIII. SATURDAY, AUGUST 7, 1920. PRICE TWOPENCE

### THE SUNDAY FUND'S SUCCESS.

By the time that The Hospital is published this week the Metropolitan Hospital Sunday Fund will have distributed £110,000, £25,000 more than the largest amount previously distributed in any year. The full facts of the year will not be available till October, but it is a fine result which deserves prompt congratulation. At the moment enough is known to encourage the belief that the supporters of the Fund are awakening to the needs of the voluntary hospitals, in the same way as the subscribers to Hospital Saturday have generally done, by deciding to double their contributions. This is encouraging, because the Sunday Fund is not capable of the same organisation as Hospital Saturday. The offertories cannot be deducted " at the source " from the incomes of the congregations, and therefore any increase is a tribute to the keenness of the clergy and their flocks. Hospital Sunday exists to emphasise the religious side of hospital work, and its success is assured when the clergy are fully alive to it. We understand that, compared with last year, the church collections show a considerable increase, and that the increase is especially noticeable in the less wealthy districts. There must be some explanation for this, and we think that two forces have been at work. Good results derive their origin from some active personality in the background, and there is no doubt that much is due to Mr. Holland-Martin, C.B., who has been one of the best Vice-Presidents that the Hospital Sunday Fund has had yet. His conviction in the face of doubts and difficulties has been invaluable, and to this we must largely attribute the realisation of the ancient hope that the Fund would raise £100,000 a year in London. After long delay that hope has been more than fulfilled in 1920.

That this sum may continue to be realised one must trust not only Mr. Holland-Martin, but, perhaps, the very difficulties in his way; for the newspaper scare earlier in the year advertised the difficulties of the voluntary hospitals in London as no appeal has ever done, and the cry of " failure " came rather from the newspapers than from their readers. The war, which invoked the State in many directions, gave the public direct experience of State machinery, and converted many of its former advocates into critics of bureaucratic institutions. Whatever are the defects of the voluntary hospitals, and the insufficiency of their accommodation is the chief, they have not made enemies of those whom they are designed to benefit. The hospitals have only to be compared with the Ministry of Pensions, which is doing much the same kind of work with infinitely greater resources, for the difference to be seen. The same bitter experience has been gained under the Insurance Act, and the working of sanatorium benefit has proved that the long purse of the State cannot supply the needs of human beings, because their first need is human treatment, and not the rigid regimentation which is all that a Government Department will supply. Without straining the comparison, we believe that the lesson has been learnt, and that, were the issue once reached, those classes who use the voluntary hospitals would make a great effort rather than exchange them for any State or municipal hospital system. If the present record figure is a sign of this reaction a useful lesson will have been taught by the recent crisis and the war.

Besides this, Hospital Sunday has had difficulties this year which, in the absence of renewed vitality, might have diminished, instead of increasing, its results. Only a few days before Hospital Sunday Alexandra Day was held, and the appeals of St. Bartholomew's and the London Hospitals were in some sense rivals with it. Yet the report of the Distribution Committee states that a sum of £111,000 is in hand. It will be interesting to examine the complete figures, to see how far the total has been gained by the outside collections and other means which the Committee of the Fund has been considering. In the case of Hospital Sunday such an examination is particularly necessary, for the Fund, of its very nature, is liable to fluctuations, and times are too serious to admit of a setback.

To prevent it we must see how the present record has been established, and for that the complete figures are required. All that we can do at present is to congratulate the Fund and Mr. Holland-Martin upon the result, for many thought that the collection of £100,000 in one year was a dream which would have to be postponed indefinitely. The year which has seen £100,000 exceeded by Hospital Sunday will always be memorable, and it will be agreed that no year could be more opportune than 1920 for this ambition to have been more than achieved. It will, we are confident, not be regarded as a goal but simply as marking a definite stage in the Fund's progress and development.

